# A case-control study of occupational magnetic field exposure and Alzheimer's disease: results from the California Alzheimer's Disease Diagnosis and Treatment Centers

**DOI:** 10.1186/1471-2377-7-13

**Published:** 2007-06-09

**Authors:** Zoreh Davanipour, Chiu-Chen Tseng, Pey-Jiuan Lee, Eugene Sobel

**Affiliations:** 1 Department of Neurology, Keck School of Medicine, University of Southern California, Los Angeles, CA 90033, USA; 2 Department of Preventive Medicine, Keck School of Medicine, University of Southern California, Los Angeles, CA 90033, USA

## Abstract

**Background:**

A few studies have investigated a possible relationship between Alzheimer's disease (AD) and occupations with extremely low frequency magnetic field (MF) exposure. The purpose of this study was to further evaluate this possible association in a large patient population with expert diagnoses.

**Methods:**

Subjects came from the 8 of the 9 California Alzheimer's Disease Diagnostic and Treatment Centers not previously used in an earlier study. Cases had probable or definite AD; controls primarily had a dementia-related problem other than vascular dementia (VaD) and some were not demented upon expert examination. Occupations were classified as having low, medium or high MF exposure, based upon previous research, replicating the exposure methodology used in our previous published studies.

**Results:**

Occupational information was available for 98.6% of the 1527 cases and 98.5% of the 404 controls with age-at-initial examination known to be at least 65. Among cases, 2.1% and 5.4% had high and medium occupational MF exposure, respectively, while among controls the percentages were 0.8% and 3.0%. In univariate analyses, the odds ratio (OR) for subjects with medium or high MF exposures combined was 2.1 (p < 0.01), while for high exposure alone the OR was 2.9 (p < 0.08). Two models were used in multivariate analyses, with gender, stroke, and either age-at-onset or age-at-initial examination as covariates. The ORs for MF exposure varied little between the two models: 2.2 (p < 0.02) and 1.9 (p < 0.03) for medium or high exposure; 2.7 (p < 0.11) and 3.2 (p < 0.12) for high exposure. OR estimates for females were higher than for males, but not significantly higher. There were no material differences between the ORs resulting from univariate and multivariate analyses.

**Conclusion:**

Elevated occupational MF exposure was associated with an increased risk of AD. Based on previous published studies, the results likely pertain to the general population.

## Background

Alzheimer's disease (AD) is the 8^th ^leading cause of death, considering all age groups, in the United States [1]. AD is also a serious problem for the families of AD patients in terms of the significant stresses of caregiving and financial costs. The annual cost to businesses has been estimated to be $61 billion dollars in 2002, including absenteeism, productivity, and employee replacement resulting from caregiving, health, and longterm care expenditures [2]. To date, only genetic mutations for early-onset AD have been identified as risk factors, and these relate to only 5% – 10% of all AD cases. The e4 allele of the apolipoprotein E gene is known to be a susceptibility factor for late-onset AD [3]. No environmental, occupational, or lifestyle risk factors have been firmly identified. The identification of causes not related to genetic mutations, effective screening assays and tests, delaying marked clinical symptoms, and effective treatment are important public health goals.

In 1996, Sobel *et al*. published findings supporting an association between longterm exposure to power frequency (ELF, 50–60 Hz) magnetic fields (MF) and the incidence of Alzheimer's disease using data from one of the State of California's Alzheimer's Disease Diagnosis and Treatment Center (ADDTC) programs (Rancho Los Amigos, Los Angeles) [4]. This work was suggested by the initial 1995 findings by Sobel *et al *[5]. We have now had the opportunity to greatly extend the 1996 study of the single ADDTC site to the other eight (8) sites, using data and forms housed at the ADDTC's Data Coordinating Center at the University of California San Francisco.

## Methods

The University of Southern California Institutional Review Board (IRB) approved this study. No informed consent was necessary as this study used existing data which was obtained with IRB approval and informed consent.

### ADDTC sites and occupational data availability

Eight (8) of the nine California ADDTC sites provided data for this study. The 9^th ^site, Rancho Los Amigos (RLA) in Los Angeles, was not used because their patients were the basis of in an earlier published study [4]. The sites in this study are Berkeley, Fresno, Irvine, Los Angeles, Palo Alto, Sacramento, San Diego, and San Francisco. These sites are operated by the University of California (UC) Davis (2), UC Irvine, UC San Diego, UC San Francisco (2), Stanford University, and the University of Southern California. All diagnoses were made by expert neurologists or psychiatrists according to NINCDS-ADRDA criteria [6,7]. All patients diagnosed at each of the eight ADDTC sites for whom data forms were completed and sent to the Data Coordinating Center at the University of California San Francisco through 1998 were included in the study. There may have been a few patients diagnosed close to the end of 1998 for whom data forms were not yet sent to the coordinating center. All subjects meeting the *a priori *diagnostic and age criteria, age 65+ at initial examination, were included in the study.

Data forms were all initially sent to the Coordinating Center at UC San Francisco. Later, patient data were computerized locally at some of the sites and then sent electronically to the coordinating center. Consequently, the Coordinating Center did not have the actual forms for all patients. The electronic dataset only contains already categorized occupational information. Because the occupational categories used by the Coordinating Center were broad, they are not appropriate for use in determining likely magnetic field exposure. Thus, occupational information needed to be obtained directly from the forms, which were only obtained from the Coordinating Center due to funding limitations. Availability of these forms is independent of diagnosis and occupation within each site.

### Case-control criteria

All subjects aged 65 or older at their initial ADDTC examination were eligible for this study. This age cut-off was used to lower the number of AD subjects whose disease was primarily genetic in origin. Table 1 provides the specific diagnoses for case or control designation. The same criteria for designation of cases and controls used in Sobel *et al*. [4] was used in this study. Subjects with a diagnosis of vascular dementia (VaD) or mixed (AD-VaD) dementia were specifically excluded as controls because VaD may also be associated with MF exposure.

**Table 1:** Case and Control Diagnoses

Diagnoses	Number of Patients
Case subjects (n = 1763)	
Probable or definite AD	1763
Control subjects (n = 543)	
1. Alcohol abuse or dependence/current alcohol use current alcohol use	15
2. CNS infection	2
3. Cognitive impairment/dementia due to age-associated memory impairment	91
4. Dementia, specific diagnosis deferred	112
5. Frontal lobe degeneration	7
6. Lewy body disease	31
7. Medication (toxic effect or metabolic derangement/metabolic disorder/toxin)	15
8. No disease	20
9. Normal pressure hydrocephalus	3
10. Not demented	3
11. Parkinson's disease	32
12. Pick's disease/other frontal temporal syndrome	10
13. Progressive supranuclear palsy	5
14. Pseudodementia	119
15. Other definitive diagnoses (non-AD, non-VaD, not mixed AD/VaD)	78

### Magnetic field exposure criteria

Occupational information on the "primary" occupation was requested on the ADDTC data form. Because many of the stated occupations would have required promotions or advancement (e.g., pilot, office manager, personal investments, film reviewer), the "primary" occupation was certainly at times the last or near the last occupation. The form requested occupational title and a description of the tasks carried out. The criteria for designation of an occupation as having likely high or medium magnetic field exposure are based on field measurements and are the same as used in the previous studies by Sobel *et al*., except pilots are now included in the exposed group based on measurements taken by one of us (ES) [4,5]. We primarily used the MF exposure data obtained by Bowman *et al*. [8] and Hansen *et al*. [9] to classify individual occupations. The occupational page from each form at the coordinating center was photocopied and reviewed for likely medium or high (M/H) magnetic field exposure (by ES, who was blinded as to case-control status). The occupations designated as having high MF exposure were those previously determined as likely to result in average (geometric mean) exposure over 10 milligauss (mG) or regular intermittent exposure over 100 mG. Occupations designated as having medium exposure were those previously found as likely to result in average (geometric mean) exposure between 2 and 10 mG or regular intermittent exposure over 10 mG. (Note that 1 mG = 0.1 microtesla (μT), the European and Système International d'Unités unit of measurement.) We emphasize that each individual working a medium occupation was likely to receive an average MF exposure over 2 mG or regular intermittent exposure over 10 mG during each work day. This is different than measuring a sample of workers over a work day and judging the average exposure to be over 2 mG, a criterion used in many published studies. For example, approximately 50% of workers in an occupation with average exposure over 2 mG will have a personal average below 2 mG.

The specific occupations classified as likely having high or medium MF exposure are provided in Table 2. The listed specified tasks related to each subject's "primary" occupation were used to insure that the subject's occupation as listed conformed with the tasks of the workers whose MF exposure was measured in Bowman *et al*. [8] and Hansen *et al*. [9], or of a pilot. Housewife and homemaker were classified as low MF exposure occupations. We note that there was no information concerning the home environment and hobbies, e.g., sewing.

**Table 2:** Occupations Classified as Likely Associated with Medium and High MF Exposure

Occupation	No. of Case and Control Subjects (a, b)*
**High MF Exposure**	
Pilot	(2, 0)
Sewing machine operator/clothing cutter/dressmaker	(24, 3)
Welder	(6, 0)
SUB-TOTAL	**(32, 3)**
**Medium MF Exposure**	
Carpenter, carpenter & machinist	(8, 1)
Appliance repair	(1, 0)
Aviator/pilot (perhaps not always in the pilot seat)	(5, 1)
Welder (part-time)	(1, 0)
Engineer (broadcast)	(1, 0)
Telephone operator, switchboard operator police dispatcher	(10, 3)
Computer operator	(2, 0)
Sewing machine operator (part-time)	(8, 0)
Worked in apparel manufacturing (not necessarily sewing machine operator)	(3, 0)
Electrician, electrical engineer, electrical mechanic, electrical assembler	(16, 3)
Electronic engineer, electronic assembler, electronic technician, electronics worker	(6, 1)
Forklift operator	(1, 0)
Furniture maker/manufacturer	(1, 0)
Machinist	(9, 3)
Metal fabricator/sheet metal worker	(4, 0)
Railroad worker, railroad brakeman	(2, 0)
Tool maker, tool maker & machinist	(3, 0)
SUB-TOTAL	**(81,12)**
TOTAL	**(113,15)**
PERCENT OF SUBJECTS	**(7.5%,3.8%)**

* (a, b): a = case number; b = control number

### Statistical analyses

Logistic regressions to estimate (OR) ratios and trends were performed using SAS [10]. For multivariate logistic regression, stepwise, forward inclusion of variables was used in model building, with a p-value of 0.05 to enter or remain in the model, except for MF exposure which was required to be in the model. Age-at-onset and age-at-exam were not considered simultaneously in model building. Rather one or the other was considered separately. The covariate candidates for inclusion were those statistically significant in univariate analyses. For the multivariate analyses with females only, we included history of stroke for consistency, even though not significant, because this variable was statistically significant for analyses with females and males combined and for males only.

Age-at-onset and age-at-exam were used for modeling only to determine whether they could affect the odds ratio estimates for MF exposure. Age-at-onset is a somewhat nebulous concept for which there is no effective or accepted operational definition. It is of necessity determined retrospectively from the opinion of close relatives or friends of the subject. Age-at-exam clearly depends on the seriousness of the perceived dementia and other factors, such as insurance and the availability of caregiving. Inclusion of either of these variables could possibly influence the odds ratio estimates for MF exposure.

## Results

### Background

Seventeen hundred sixty three (1763) ADDTC subjects were diagnosed as having AD, while 543 subjects were not so diagnosed. Among the 1763 cases, 151 (8.6%) were missing their age-at-initial examination (subsequently referred to as age-at-exam) and 85 (4.8%) were below age 65 at their examination. A further 1.4% (n = 25) were missing occupational information. Thus, there were 1502 cases available for analysis. Among the 543 controls, 50 (9.2%) were missing their age-at-exam and 89 (16.4%) were below age 65 at their examination. A further 1.5% (n = 8) were missing occupational information. Thus, there were 396 controls available for analysis. Thus there were 1502 cases and 396 controls available for analyses. Thirty-two (32, 2.1%) of the cases and 3 (0.8%) of the controls had an occupation classified as being associated with high MF; 81 (5.4%) of the cases and 12 (3.0%) of the controls had an occupation classified as being associated with medium MF (Table 2).

Table 3 provides demographic and other statistics by case-control status. Females constituted 71.4% of the cases and 62.9% of the controls (p < 0.002). Mean age-at-exam and mean age-at-symptom onset (subsequently referred to as age-at-onset) were statistically significantly different between cases and controls (p < 0.001 and p < 0.003, respectively), but were still rather close. The ordinalized distributions (3 classes for age-at-diagnosis and 4 classes for age-at-symptom onset) were statistically significantly different (p < 0.001 and p < 0.005, respectively). Ethnicity, education, current income, and history of stroke were also statistically different between cases and controls. The percentages of cases and controls with a history of smoking were very similar and not statistically different. The distributions of low, medium, and high MF occupations, for females and males combined and for females, were different between cases and controls (p < 0.03). For males, the distributions were not significantly different.

**Table 3:** Descriptive Statistics: Subjects with Occupational Information*

Variable	Classification	Case	Control	p-Value
Gender	Females	1073 (71.4)	249 (62.9)	< 0.002
	Males	429 (28.6)	147 (37.1)	
Age-at-exam	65–74	450 (30.0)	184 (46.5)	< 0.001
	75–84	801 (53.3)	176 (44.4)	
	85+	251 (16.7)	36 (9.1)	
	Mean (SD)	78.2 (6.4)	75.6 (6.6)	< 0.001
Age-at-onset	< 65	141 (9.7)	52 (15.6)	< 0.005
	65–74	665 (46.0)	162 (48.5)	
	75–84	569 (39.3)	105 (31.4)	
	85+	71 (4.9)	15 (4.5)	
	Missing	56	62	
	Mean (SD)	73.4 (7.2)	72.0 (7.3)	< 0.003
Ethnicity	White	1163 (77.6)	284 (71.7)	< 0.02
	Hispanic	168 (11.2)	65 (16.4)	
	Other	168 (11.2)	47 (11.9)	
	Missing	3	0	
Education	0–5	102 (6.9)	33 (8.5)	< 0.05
	6–11	422 (28.7)	87 (22.3)	
	12+	949 (64.4)	270 (69.2)	
	Missing	29	6	
Current income	< $10,000	448 (39.1)	127 (41.8)	= 0.05
	$10,000 – $19,999	367 (33.0)	73 (24.0)	
	$20,000 +	332 (28.9)	104(34.1)	
	Missing	355	92	
Hx of smoking	Yes	547 (37.7)	148 (38.7)	NS^^^: > 0.71
	No	903 (62.3)	234 (61.3)	
	Missing	52	14	
Hx of stroke	Yes	103 (7.1)	43 (11.4)	< 0.02
	No	1347 (92.9)	335 (88.6)	
	Missing	52	18	
MF exposure level	High	32 (2.1)	3 (0.8)	< 0.03
	Medium	81 (5.4)	12 (3.0)	
	Low	1389 (92.5)	381 (96.2)	

Female Only				
MF exposure level	High	19 (1.8)	1 (0.4)	< 0.03
	Medium	61 (5.7)	6 (2.4)	
	Low	993 (92.5)	242 (97.2)	
Male Only				
MF exposure level	High	13 (3.0)	2 (1.4)	NS: > 0.50
	Medium	20 (4.7)	6 (4.1)	
	Low	396 (92.3)	139 (94.6)	

* Figures in parentheses are percentages, unless otherwise noted.

^^ ^NS = Not Significant

### Univariate Odds Ratio analyses for MF exposure

Table 4 provides univariate odds ratio analyses for MF exposure and the other variables in Table 3. For males and females combined, the OR for medium or high MF exposure was 2.1, p < 0.01, 95% confidence interval (CI) = 1.2 – 3.9, while the OR for high MF exposure was 2.9, (95% CI = 0.9 – 9.6). The p-value was 0.08, perhaps due to the relatively small proportion of subjects with a high MF occupation. For females, the OR for medium/high MF exposure was 2.8, p = 0.01, 95% CI = 1.3 – 6.1. The OR for high MF exposure was 4.6, p > 0.13, 95% CI = 0.6 – 34.8. For males, the OR for medium/high MF exposure was 1.4, p > 0.35, 95% CI = 0.7 – 3.2. The OR for high MF exposure was 2.3, p > 0.2, 95% CI = 0.5 – 10.2.

**Table 4:** Univariate Analysis of MF Exposure and Other Variables

Variable	Level	Odds Ratio (95% CI)	p-value
Female + Male			
MF Exposure	Low	1.0	Trend: < 0.01
	Medium	1.9 (1.0 – 3.4)	0.05
	High	2.9 (0.9 – 9.6)	< 0.08
	High/Medium	2.1 (1.2 – 3.9)	< 0.01
Female Only			
MF Exposure	Low	1.0	Trend: < 0.01
	Medium	2.5 (1.1 – 5.8)	< 0.04
	High	4.6 (0.6 – 34.8)	NS^^^: > 0.13
	High/Medium	2.8 (1.3 – 6.1)	= 0.01
Male Only			
MF Exposure	Low	1.0	Trend: NS: >0.25
	Medium	1.2 (0.5 – 3.0)	NS: > 0.7
	High	2.3 (0.5 – 10.2)	NS: > 0.2
	High/Medium	1.4 (0.7 – 3.2)	NS: > 0.35

Female + Male			
Gender	Males	1.0	
	Females	1.5 (1.2 – 1.9)	= 0.001
Age-at-Exam	65–74	1.0	Trend: < 0.0001
	75–84	1.9 (1.5 – 2.4)	< 0.0001
	85+	2.9 (1.9 – 4.2)	< 0.0001
Age-at-Onset	< 65	1.0	Trend: < 0.002
	65–74	1.5 (1.05 – 2.2)	< 0.03
	75–84	2.0 (1.4 – 2.9)	< 0.001
	85+	1.7 (0.9 – 3.3)	< 0.09
Education	1 = 0–5 Years	1.0	
	per "Unit"^#^	1.1 (0.9 – 1.3)	NS: > 0.35
Ethnicity	White	1.0	
	Hispanic	0.6 (0.5 – 0.9)	< 0.01
	Other	0.9 (0.6 – 1.2)	NS: > 0.4
Income	1 = < $5,000	1.0	
	per "Unit"*	0.9 (0.8 – 1.05)	NS: > 0.2
Smoking	No	1.0	
	Yes	0.96 (0.8 – 1.2)	NS: > 0.7
Stroke	No	1.0	
	Yes	0.6 (0.4 – 0.9)	< 0.01

* Units: 2 = $5000–$9999; 3 = $10000–$19999; 4 = $20000–$34999; 5 = ≥ $35000.

^# ^Units: 2 = 6–11 Years; 3 ≥ 12 Years.

^NS = Not Significant

Trend analyses for the ORs for low, medium, high MF occupations were also conducted (Table 4). For the females and the men and women combined the trends were statistically significant, while for the males the trend was not significant.

### Multivariate Odds Ratio analyses

Multivariate, forward stepwise inclusion analyses using logistic regression were conducted for males and females combined because of possible confounding. All variables in Table 3 were eligible for inclusion in the final models for the females and males combined (Table 5). When medium and high MF occupations were combined, M/H magnetic field exposed occupations, gender, stroke, and either age-at-exam or age-at-onset (depending upon which was in the model) were significant risk factors. The M/H magnetic field exposed occupational ORs were 2.2 (p < 0.02; 95% CI: 1.2 – 3.9) and 2.0 (p < 0.03; 95% CI: 1.1 – 3.7), respectively. When only high MF occupations were considered (versus low MF occupations), the same covariates were significant. However, the ORs for high MF occupations were larger (2.8 and 3.3 for age-at-exam and age-at-onset, respectively), while the significance levels were below 0.10 and 0.11, respectively, perhaps due to the small relative frequency of high MF exposure. The corresponding 95% CIs were, respectively, (0.8 – 9.3) and (0.8 – 14.1). Thus, the multivariate MF OR point estimates were essentially unchanged from the univariate results.

**Table 5:** Multivariate Models of MF Exposure and Other Risk Factors/Confounders: Females and Males Combined

Risk factor	Odds ratio	95% CI	p-value
High/Medium MF	2.2	(1.2 – 3.9)	< 0.01
Gender (females = 1; males = 0)	1.4	(1.1 – 1.8)	< 0.004
Age-at-exam per unit*	1.8	(1.5 – 2.2)	< 0.0001
Stroke	0.6	(0.4 – 0.9)	< 0.008

High MF	2.8	(0.8 – 9.3)	< 0.10
Gender (females = 1; males = 0)	1.4	(1.1 – 1.8)	< 0.01
Age-at-exam per unit*	1.8	(1.5 – 2.2)	< 0.0001
Stroke	0.6	(0.4 – 0.9)	< 0.01

High/Medium MF	2.0	(1.1 – 3.7)	< 0.03
Gender (females = 1; males = 0)	1.5	(1.1 – 1.9)	< 0.003
Age-at-onset per unit	1.3	(1.1 – 1.5)	< 0.003
Stroke	0.6	(0.4 – 0.97)	< 0.02

High MF	3.3	(0.8 – 14.1)	NS^^^: > 0.10
Gender (females = 1; males = 0)	1.4	(1.1 – 1.9)	< 0.01
Age-at-onset per unit	1.3	(1.1 – 1.5)	< 0.04
Stroke	0.6	(0.4 – 0.9)	< 0.01

* Units:

Age-at-exam: 1 = 65–74; 2 = 75–84; 3 = 85+

Age-at-onset: 0 = 65-; 1 = 65–74; 2 = 75–84; 3 = 85+

^^ ^NS = Not Significant

Multivariate analyses were also conducted for females and for males separately (Tables 6 and 7). For females, medium/high EMF had significant ORs, 3.3 and 2.9, for using age-at-exam and age-at-onset. The ORs for high EMF were 4.0 and 3.2, respectively, but were not statistically significant. For males, the ORs for medium/high EMF, 1.4 and 1.3, and for high EMF, 2.1 and 3.5, were not statistically significant. The differences between the corresponding ORs for males and females are not statistically significant.

**Table 6:** Multivariate Models of MF Exposure and Other Risk Factors/Confounders: Females Only

Risk factor	Odds ratio	95% CI	p-value
High/Medium MF	3.3	(1.3 – 8.4)	< 0.02
Age-at-exam per unit*	1.9	(1.5 – 2.3)	< 0.0001
Stroke	0.7	(0.4 – 1.1)	NS^^^: > 0.11

High MF	4.0	(0.5 – 30.5)	NS: > 0.17
Age-at-exam per unit	1.8	(1.5 – 2.3)	< 0.0001
Stroke	0.7	(0.4 – 1.1)	NS: > 0.13

High/Medium MF	2.9	(1.1 – 7.3)	< 0.03
Age-at-onset per unit*	1.3	(1.03 – 1.6)	< 0.03
Stroke	0.7	(0.4 – 1.2)	NS: > 0.20

High MF	3.2	(0.4 – 24.4)	NS: > 0.25
Age-at-onset per unit	1.3	(1.02 – 1.5)	< 0.04
Stroke	0.7	(0.4 – 1.3)	NS: > 0.25

* Units:

Age-at-exam: 1 = 65–74; 2 = 75–84; 3 = 85+

Age-at-onset: 0 = 65-; 1 = 65–74; 2 = 75–84; 3 = 85+

^^^NS = Not Significant

**Table 7:** Multivariate Models of MF Exposure and Other Risk Factors/Confounders: Males Only

Risk factor	Odds ratio	95% CI	p-value
High/Medium MF	1.4	(0.3 – 1.6)	NS^^^: > 0.41
Age-at-exam per unit*	1.7	(1.2 – 2.3)	< 0.001
Stroke	0.5	(0.3 – 0.9)	< 0.03

High MF	2.1	(0.5 – 10.0)	NS: > 0.31
Age-at-exam per unit	1.7	(1.3 – 2.4)	< 0.001
Stroke	0.5	(0.3 – 0.8)	< 0.02

High/Medium MF	1.3	(0.6 – 3.1)	NS: > 0.50
Age-at-onset per unit*	1.4	(1.02 – 1.8)	< 0.04
Stroke	0.5	(0.3 – 0.9)	< 0.02

High MF	3.5	(0.4 – 27.5)	NS: > 0.23
Age-at-onset per unit	1.4	(1.02 – 1.8)	< 0.04
Stroke	0.4	(0.2 – 0.8)	< 0.01

* Units

Age-at-exam: 1 = 65–74; 2 = 75–84; 3 = 85+

Age-at-onset: 0 = 65-; 1 = 65–74; 2 = 75–84; 3 = 85+

^^ ^NS = Not Significant

## Discussion

This study has a number of strengths: the sample size was large; the finding of dementia and its differential diagnosis was made by experts following nationally agreed upon criteria; elimination of subjects whose examination was prior to age 65 lowered the number of AD cases primarily of genetic origin; the occupational information included job title and a description of the duties of the job; the staff collecting the data had no idea that one day the data may be used to study MF exposure as a risk factor; the study sites were spread across California, but used a single protocol; and the criteria for medium and high MF exposure used field measurements, was conservative, and were the same as in the previous Sobel *et al*. studies [4,5]. The relative frequencies of high and medium MF exposure were rather low because of the conservative nature of the criteria: 1.8% high and 4.9% medium.

The OR estimates are unlikely to be biased upwards, but may certainly be biased towards 1.0. We have taken hundreds of MF measurements of sewing machines, operators using both industrial and home sewing machines, and the ambient MF levels in the apparel manufacturing environment. We have also taken several measurements of the MF exposure of pilots and welders. These were the only occupations of the subjects which were classified as having high MF exposure. There is really no argument that the 35 subjects in the high MF classification most likely had high and longterm MF exposure. There is more room for error in the medium MF classification, however. Some of these 93 subjects may have had low MF exposure, but it is unlikely that they had high exposure. Among the 1770 study subjects classified as having low MF exposure (Table 3), some may have had medium or even high MF exposure because there are many, perhaps somewhat uncommon or perhaps even common, reasons for extended high MF exposure, e.g., sitting very close to AC/DC transformers which are common in an office environment, having an office next to a communications equipment room, sleeping on the other side of a wall with a circuit breaker box, spending significant time in a kitchen with a microwave oven operating or using a handheld mixer, blow drying hair (e.g., dog's hair), using leaf blowers carried as backpacks, operating a car with certain electronic or electrical equipment actually near the driver's seat. These errors in classification all would tend to bias the OR estimators towards 1.0.

The use of demented subjects as controls may seem somewhat problematic. The initial Sobel *et al*. study [5], included 3 independent studies with varying controls: subjects hospitalized for an illness other than a neurologic problem; non-demented "neighborhood" subjects; patients with vascular dementia. The odds ratio estimates, for both females and males combined, were 2.9, 3.0, and 3.1 for M/H exposure. The combined sample odds ratio was 3.0 (p < 0.001). In the second Sobel *et al*. study [4], the controls were precisely as defined in the current study and the initial study. The M/H exposure odds ratio estimate was 3.9 (p = 0.006). A population-based study from Turkey by Harmanci *et al*. [11] used the same methodology as in our studies and expert diagnoses. The M/H odds ratio estimate was 4.0 (p < 0.05). In current study, the univariate M/H odds ratio was 2.2 (p < 0.02).

Three other studies have been published which used expert diagnoses. The Feychting *et al*. study [12] was small, used 2.0 mG as the cutpoint for M/H exposure, and had two control groups. They found odds ratios of 2.4 and 2.7 (p > 0.05). The Qui *et al*. study [13] also used 2.0 mG as the cutpoint, a Swedish "job exposure matrix" and expert diagnoses. They found an odds ratio of 0.9 or 1.0 (p > 0.05), depending upon the type of statistical adjustment. However, the odds ratio for males was 2.3 (p < 0.05) and for females was 0.8 (p > 0.05). Qui *et al*. classified seamstresses who used a home sewing machine as having low exposure. In addition, 23.8% of the females in the study were classified as having M/H exposure, as opposed to 3.3% in the current study. Qui *et al*. certainly classified some occupations as having M/H exposure which we classified as low exposure. They did not provide any information, except for seamstresses and telephone operators, as to which occupations were classified as M/H exposed.

Contrary to the statements in the Qui *et al*. study, the classification of occupations in the Sobel *et al*. studies [4,5] and the current study were based on extensive occupational measurements, which is particularly true for seamstresses. Graves *et al*. [14] studied unionized workers and their families who subscribed to a large HMO. AD cases were expertly diagnosed. Their study was small and certainly found no relationship between MF exposure and AD. However, their operational definition of exposure was unusual and resulted in more than 19% of the subjects being classified as exposed. There were no seamstresses or tailors in their study, probably because workers in these occupations are seldom in unions.

The other published studies on AD and MF exposure used death certificate or hospital discharge records to determine AD status and were thus primarily not based on expert diagnosis. Among these studies, 4 had somewhat positive aspects [15-18] and 3 were negative [19-21]. Because other dementias are very often misdiagnosed as AD by community-based physicians, death certificate and even hospital discharge records are often incorrect when AD is provided as a cause of death or hospitalization. In addition, AD, when present, is often not specified as a cause or contributing cause of death. These two errors will bias odds ratio estimators towards 1.0.

The current study also has weaknesses. These include the following: occupational information would have been more detailed if MF exposure had been of interest, with interviewers and subjects blinded as to the study hypothesis; information on the use of equipment generating magnetic fields in hobbies or housework was not collected; lifetime occupational information was not collected; subjects were not sampled from a well-defined population; ApoE genotyping was not performed; specific measurements of exposure were not conducted for study subjects. Future studies can overcome these weaknesses if funding becomes available.

## Conclusion

We conclude the following.

1. The varying nature of the control subjects, the timing and geographic differences in the conduct of the studies, the collection of occupational information without any knowledge that the data would be used for studies of occupational MF exposure, the expert diagnoses of AD, and the similarity of the odds ratio estimates all argue for a lack of bias away from the null in the studies conducted by Sobel *et al*. [4,5] and the current study.

2. Other studies, but not all other studies, indicate that working in occupations with high occupational exposure to magnetic fields over a substantial time period may be a risk factor for Alzheimer's disease [11-21].

3. Combining the 5 studies in the Sobel *et al*. [4,5] and current papers, the Mantel-Haenszel odds ratio for sewing machine operators (seamstresses, dressmakers, tailors) is 3.7, p < 0.001. This indicates that individuals in this occupation are likely to be at increased risk of developing AD, for some reason. It is important to confirm this statement with targeted studies and, if confirmed, to determine the etiologically relevant exposure(s).

4. Firm identification of occupational and environmental exposures causing AD may permit a deeper understanding of the biological processes leading to AD. This knowledge may then be useful in developing prevention strategies, screening assays, and early effective treatment.

## Abbreviations

Aβ, amyloid beta

AD, Alzheimer's disease

ADDTC, Alzheimer's Disease Diagnosis and Treatment Center

CI, confidence interval

ELF, extremely low frequency

Hz, Hertz

MF, magnetic field

mG, milligauss

M/H, medium or high

OR, odds ratio

RLA, Rancho Los Amigos

VaD, vascular dementia

## Competing interests

The author(s) declare that they have no competing interests.

## Authors' contributions

ZD & ES contributed to all aspects of the design, implementation, analyses, and interpretation of the study, drafts, revisions and critical review of the paper. C-CT & P-JL contributed to the data management and data analyses.

## Pre-publication history

The pre-publication history for this paper can be accessed here:


